# Microfluidic co-culture of pancreatic tumor spheroids with stellate cells as a novel 3D model for investigation of stroma-mediated cell motility and drug resistance

**DOI:** 10.1186/s13046-017-0654-6

**Published:** 2018-01-12

**Authors:** Ji-Hyun Lee, Seul-Ki Kim, Iftikhar Ali Khawar, Su-Yeong Jeong, Seok Chung, Hyo-Jeong Kuh

**Affiliations:** 1 Department of Biomedicine & Health Sciences, College of Medicine, The Catholic University of Korea, Seoul, Republic of Korea; 2 School of Mechanical Engineering, College of Engineering, Korea University, Seoul, Republic of Korea; 3 Department of Medical Life Sciences, College of Medicine, The Catholic University of Korea, 222 Banpo-daero, Seocho-ku, Seoul, 06591 Republic of Korea

**Keywords:** Microchannel plate, EMT, Pancreatic cancer, Cancer-Stroma co-culture, Tumor microenvironment

## Abstract

**Background:**

Pancreatic stellate cells (PSCs), a major component of the tumor microenvironment in pancreatic cancer, play roles in cancer progression as well as drug resistance. Culturing various cells in microfluidic (microchannel) devices has proven to be a useful in studying cellular interactions and drug sensitivity. Here we present a microchannel plate-based co-culture model that integrates tumor spheroids with PSCs in a three-dimensional (3D) collagen matrix to mimic the tumor microenvironment in vivo by recapitulating epithelial-mesenchymal transition and chemoresistance.

**Methods:**

A 7-channel microchannel plate was prepared using poly-dimethylsiloxane (PDMS) via soft lithography. PANC-1, a human pancreatic cancer cell line, and PSCs, each within a designated channel of the microchannel plate, were cultured embedded in type I collagen. Expression of EMT-related markers and factors was analyzed using immunofluorescent staining or Proteome analysis. Changes in viability following exposure to gemcitabine and paclitaxel were measured using Live/Dead assay.

**Results:**

PANC-1 cells formed 3D tumor spheroids within 5 days and the number of spheroids increased when co-cultured with PSCs. Culture conditions were optimized for PANC-1 cells and PSCs, and their appropriate interaction was confirmed by reciprocal activation shown as increased cell motility. PSCs under co-culture showed an increased expression of α-SMA. Expression of EMT-related markers, such as vimentin and TGF-β, was higher in co-cultured PANC-1 spheroids compared to that in mono-cultured spheroids; as was the expression of many other EMT-related factors including TIMP1 and IL-8. Following gemcitabine exposure, no significant changes in survival were observed. When paclitaxel was combined with gemcitabine, a growth inhibitory advantage was prominent in tumor spheroids, which was accompanied by significant cytotoxicity in PSCs.

**Conclusions:**

We demonstrated that cancer cells grown as tumor spheroids in a 3D collagen matrix and PSCs co-cultured in sub-millimeter proximity participate in mutual interactions that induce EMT and drug resistance in a microchannel plate. Microfluidic co-culture of pancreatic tumor spheroids with PSCs may serve as a useful model for studying EMT and drug resistance in a clinically relevant manner.

**Electronic supplementary material:**

The online version of this article (10.1186/s13046-017-0654-6) contains supplementary material, which is available to authorized users.

## Background

Pancreatic ductal adenocarcinoma (PDAC) is the fourth most common cause of cancer-related deaths worldwide. It is one of the few human malignancies having nearly 100% mortality with a median survival time of less than 6 months and 5-year survival rates of less than 4% [1]. The aggressive disease progression and resistance to conventional therapeutic regimens are the main reasons behind the poor prognosis. Currently, gemcitabine is the standard of care treatment option but it improves overall survival by only 6 months [2]. Novel therapeutic agents or regimens have not yet been developed, which may be in part attributed to a lack of appropriate models representing the in vivo tumor and its microenvironment. Despite the availability of many genetically engineered mouse models, there is an urgent need to develop physiologically relevant in vitro models for studying cancer progression as well as drug resistance.

PDAC is well known as a stroma-rich, desmoplastic type cancer in which stroma constitutes up to 90% of tumor volume [3]. Stromal components including cancer-associated fibroblast (CAFs), immune cells, extracellular matrix (ECM), as well as many soluble factors are known to stimulate the aggressiveness of PDAC [4–6]. Among these, CAFs are considered an important stromal part since they accumulate ECM and secrete soluble factors that are associated with disease progression and chemoresistance [7]. Pancreatic stellate cells (PSCs) are considered a major source of CAFs in PDAC [5, 8]. PSCs exist in a quiescent state in the normal pancreas but transform into an activated state when interacting with cancer cells [5, 8]. PSCs create fibrotic and hypoxic microenvironments and promote tumor growth and metastasis as shown in studies using in vivo models and in vitro co-cultures [4, 9]. PSCs play roles in chemoresistance in direct and indirect manners through modulating drug metabolism or sensitivity and drug delivery by excessive ECM deposition [10, 11]. Major ECM components such as fibronectin, hyaluronic acid, proteoglycans and collagen, have been targeted to improve responses to chemotherapy in mouse models and patients [4, 12]. Although PSCs may represent a promising target for drug development, several recent studies reported CAFs heterogeneity [13] and raised questions in regard to the role of PSCs and related intracellular signaling in tumor progression [10] .

The pancreatic tumor microenvironment has a critical role in tumor progression via epithelial to mesenchymal (epithelial-mesenchymal) transition (EMT) [8, 9, 14]. EMT acts as a facilitator of metastatic dissemination in the invasive margin of tumors where tumor-stroma crosstalk actively takes place [15]. EMT has also been implicated as a mechanism of drug resistance [16]. Therefore, EMT may be a potential target for anti-metastatic as well as chemosensitization strategy [17–19], which represents a demand for efficient models to study EMT.

Current in vitro models such as two-dimensional (2D) monolayer culture cannot represent tissue environment which includes cell-cell and cell-matrix interactions. It doesn’t reproduce the structural organization or pathophysiologic characteristics of carcinomas in vivo [20, 21]. Three dimensional (3D) culture models provide more in vivo like, physiologically relevant condition, so these models became important tools in cancer research and drug discovery [22, 23]. Tumor spheroids or tumoroids became popular models as they recapitulate 3D cellular organization in vivo*.* Organotypic models include culture of cells in a 3D gel of ECM material such as collagen and matrigel. As a platform for 3D cell cultures, microfluidic devices are gaining greater prominence for the study of tumor-stroma interactions, intravasation and angiogenesis [23, 24]. Microchannel structure in microfluidic devices is optimal for proximity culture of cancer cells with stromal cells and also suitable for encapsulation of tumor aggregates in the ECM. Hence, 3D cell cultures in microfluidic devices may allow in vitro study of the interactions between components of tumor microenvironment under a physiologically relevant condition [25–27].

Here we established an in vitro 3D pancreatic tumor model in a microchannel chip. Cancer cell spheroids were co-cultured with PSCs at submillimeter distance within collagen-supported microchannels. We observed that tumor spheroids and PSCs were mutually activated when co-cultured. Under co-culture condition, tumor spheroids acquired a migratory phenotype as well as drug resistance, in association with EMT changes. We suggest that our 3D tumoroid model in a microchannel chip is useful in studying cell migration, EMT, and drug resistance as well as the underlying molecular mechanisms. This model can be utilized in evaluation of therapeutic agents that could potentially modulate tumor microenvironmental interactions.

## Methods

### Cell culture

The human pancreatic cancer cell lines PANC-1, AsPC-1 and MIA PaCa-2, the human colorectal cancer cell line HT-29 were obtained from the American Type Culture Collection (ATCC). The human hepatic cancer cell line Huh-7 was obtained from the Japanese Collection of Research Bioresources Cell Bank. All cells were cultured at 37°C in a humidified atmosphere (5% CO_2_/95% air). PANC-1 and MIA PaCa-2 cells were cultured in DMEM with high glucose (Hyclone, Logan, UT, USA). AspPC-1 and HT-29 cells were cultured in RPMI-1640 medium (Gibco, Grand Island, NY, USA) and Huh-7 cells were cultured in DMEM (Gibco BRL) supplemented with 100 μg/mL streptomycin, 100 units/mL penicillin, 250 ng/mL amphotericin B and 10% fetal bovine serum (FBS, Welgene, Daegu, Korea). PSC, human pancreatic stellate cell line (HPaSteC, #3830, ScienCell, Carisbad, CA) was cultured in DMEM with high glucose (Hyclone) supplemented with 100 μg/mL streptomycin, 100 units/mL penicillin, 250 ng/mL amphotericin B and 5% heat-inactivated fetal bovine serum.

### Fabrication of PDMS microchannel plate

Microchannel plates were prepared using poly-dimethylsiloxane (PDMS; Silgard 184, Dow Chemical, Midland, MI, USA) according to the protocol reported in previous studies [28, 29]. Microchannel plates were designed to contain 4 units in a single plate. Each unit contains three cell channels for cancer and stellate cell loading in hydrogel and 4 media channels, to feed these cell channels from both sides. An SU-8 patterned master was custom-made (Amed Co., Seoul, Korea) using photolithography and then a conventional soft lithography was used on the SU-8 patterned master to produce PDMS replicas. Briefly, PDMS base and crosslinking agent were mixed thoroughly at a ratio of 10:1 (*w*/w) and poured onto the master mold and cured for 3 h at 60 °C. Upon removal from the master, inlet and outlet ports were formed using 18 G needle and 6 mm disposable biopsy punch. The open side of PDMS replicas were secured to a glass coverslip or a PDMS film (~80 μm thick) with oxygen plasma (CUTE; Femto Science, Seoul, Korea). The microchannels formed were then coated with poly-dopamine solution (2 mg/mL) to promote type I collagen adhesion onto the channel surface as previously reported [30]. Chips were then ready to be used after drying overnight in a 60 °C oven.

### 3D spheroid culture in microchannel plates

Cells were harvested during log phase of cell growth and a cell suspension was prepared. Collagen gel solution (2 mg/mL) was prepared by mixing collagen type I (rattail, BD Biosciences, San Jose, CA) with phenol red-containing PBS with the pH adjusted to 7.4 using 0.5 N NaOH. The cell suspension was mixed with the type I collagen solution at a 1:9 ratio to densities of 3 × 10^5^/ml and 5 × 10^5^/ml. Cells were loaded into each designated channel by injecting 5 μL of cell-hydrogel mixture at a density of 1.5 × 10^3^/channel for PANC-1 cells and PSCs and 2.5 × 10^3^/channel for other cancer cells. We used a 1:2 ratio of cancer cells to stellate cells as it was in the range reported to have in vivo relevancy where the ratios of 1:1 to1:3 have commonly been used [28, 31, 32]. After polymerization in a cell culture incubator for 30 mins, microchannels were filled with culture medium, and returned to the incubator for culture. We used intermittent feeding instead of constant flow. Cells were cultured for 5–10 days with media changed every day. Diameters were calculated from sectional area of spheroids measured based on bright field images using Image J and an equation assuming a circular spheroid shape (area = πr^2^). Cell aggregates with diameter larger than 40 μm were considered full spheroids.

### Labeling PANC-1 cells with cell tracker for monitoring cell migration

In order to trace the movement of cancer cells in co-culture condition, PANC-1 cells were loaded with the cell tracker PKH26 (Red fluorescent cell linker kit; Sigma-Aldrich, Saint Louis, MO, USA) before culturing in microchannel plates. The tracker staining was performed according to the manufacturer’s instructions. Briefly, cell suspensions were washed in serum free media and mixed with diluent C (supplied with the staining kit). After adding the PKH26 linker, the mixture was incubated for 3 min at room temperature, then the reaction was stopped by adding FBS. The cells were then washed 3 times with complete medium and were ready for use.

### Immunofluorescence staining and image acquisition

Expression of Ki-67, alpha smooth muscle actin (α-SMA), connective tissue growth factor (CTGF), transforming growth factor beta (TGF-β), E-cadherin, vimentin and F-actin was determined by immunofluorescence staining. Briefly, cultures in microchannels were fixed using 4% paraformaldehyde for 30 mins and further treated with 0.5% Triton X-100 for another 30 mins. After blocking non-specific binding with 5% bovine serum albumin (BSA, Affymetrix, Cleveland, OH) overnight, primary antibodies against Ki-67 (1:50, sc-15,402, Santa Cruz, Dallas, TX), α-SMA (1:50, ab5694, Abcam, Cambridge, UK), CTGF (1:50, ab6992, Abcam), E-cadherin (1:50, 3195S, Cell Signaling, Beverly, MA), vimentin (1:50, ab92547, Abcam) and F-actin (1:50, cat. no. R415, Thermo Fisher, Waltham, MA) were applied for overnight incubation at 4 °C. After incubation with Alexa Flour® 594 secondary antibody (1:2000, cat. no. Z-25307, Thermo Fisher) and DAPI (1:1000, cat. no. D9564, Sigma-Aldrich) at room temperature for 3 h, microchannels were washed with PBS and analyzed by confocal microscopy (LSM 510 Meta, Zeiss, Oberkochen, Germany). Optical sections were acquired at 6 μm intervals and stacked into a z-projection from which fluorescence intensity was determined. For quantitative comparison, data were normalized to DAPI intensity.

### Drug response assay

Viability changes were measured after exposure to anti-cancer agents such as gemcitabine, paclitaxel and oxaliplatin. PANC-1 spheroids and PSCs grown for 5 days were treated with drug or drug combinations for 72 h and stained using LIVE/DEAD reagents (BDA-1000, BIOMAX, Seoul, Korea) according to the manufacturer’s protocol. Confocal microscopic sections were acquired at 6 μm intervals and stacked into a z-projection. Fluorescence intensity was measured and an average intensity from three out of 5 fields, which covers (three fields) 80% of the effective area in each channel.

### Human proteome array analysis

The expression levels of proteins known for their roles in angiogenesis and metastasis were analyzed using the Proteome Profiler™ (Human Angiogenesis Array kit, R&D Systems, MN, USA) according to the manufacturer’s instruction. Briefly, cell lysates were prepared using a standard method and blocking of non-specific binding was done at room temperature for 1 h. Antibody array membranes were incubated with the cell lysate (1.5 mL) at 4 °C overnight and then with horseradish peroxidase-conjugated streptavidin at room temperature for 30 mins. Visualization was achieved by chemiluminescence and signal intensity was quantified using Multi Gauge V3.0 software (FUJI FILM, Japan).

### Statistical analysis

All data were expressed as the mean ± standard error (SE) of three or more independent measurements. Student's t-test as well as analysis of variance (ANOVA), followed by Tukey’s test, were used to test the statistical significance using Microsoft Excel 2010. *P* values <0.05 were considered statistically significance.

## Results

### Growth of PANC-1 tumor spheroid influenced by PSC co-culture

**Fig. 1:**
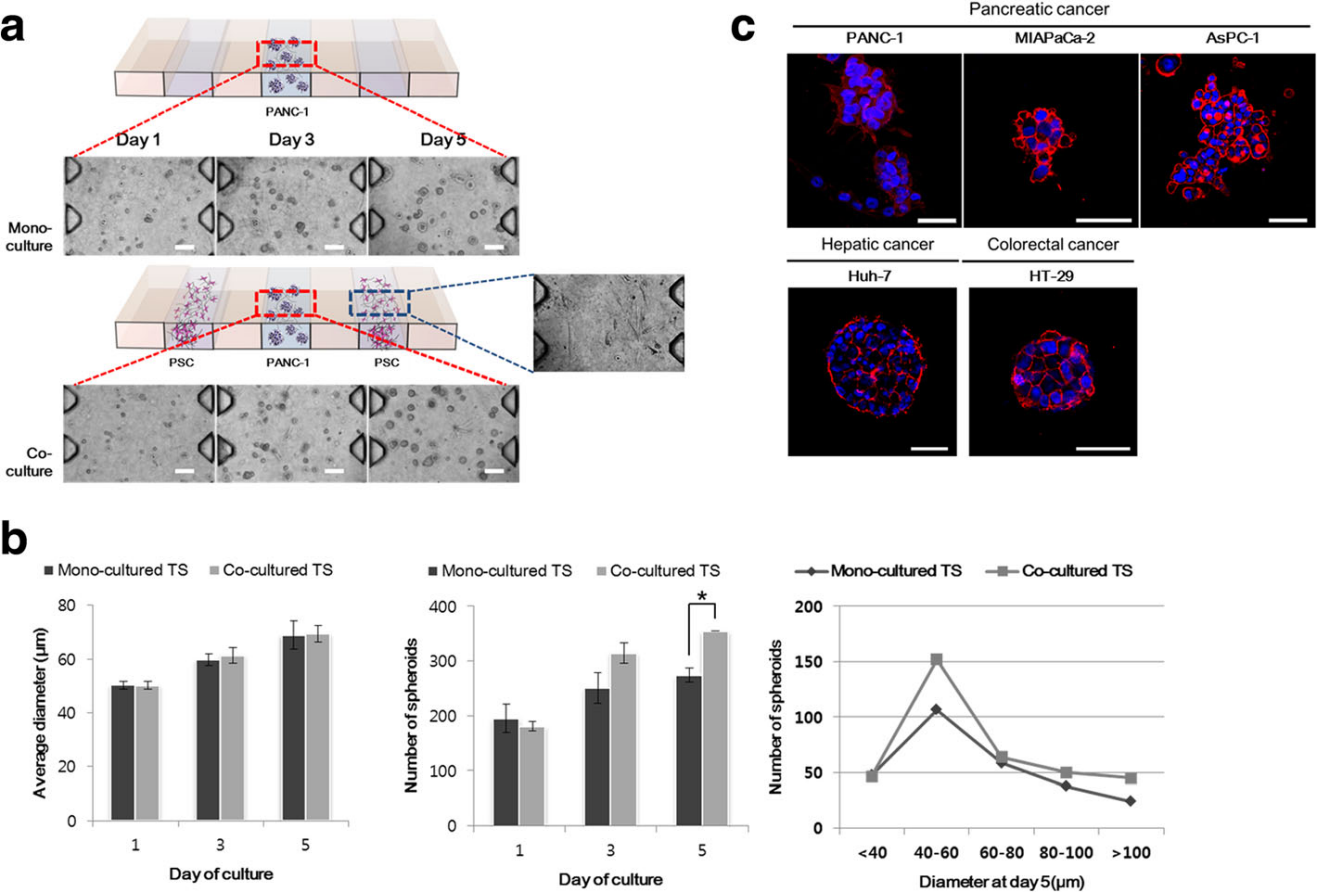
Effect of PSC co-culture on growth of PANC-1 spheroids. **a** Formation of PANC-1 spheroids in a collagen-supported microchannel plate when cultured. **b** Comparison of growth of PANC-1 spheroids over 5 days with (co-culture) or without (mono-culture) PSCs. **c** Comparison of morphology and structure of spheroids generated using pancreatic, hepatic and colorectal cancer cells. Cells were grown for 5 days in collagen-supported microchannel plates to form 3D spheroids and stained for nuclei (DAPI; blue) and F-actin (red); Scale bar, 50 μm. Cell aggregates of diameter larger than 40 μm were defined as spheroids. Optical sections were acquired at 2 μm intervals and stacked into a z-projection. Data are expressed as the mean ± SE of three independent experiments. Student’s t-test, * *p* < 0.05. PSCs, pancreatic stellate cells; TS, tumor spheroids

PANC-1 3D tumor spheroids were cultured in collagen-supported channels and their growth was monitored for 5 days under mono- and co-culture condition with PSCs (Fig. 1-a). Over the 5 days, a significant difference was observed in the number of spheroids but not in the average size of tumor spheroids between mono- and co-culture conditions. When the size distribution of tumor spheroids was analyzed, the significant difference in numbers was seen in spheroids with 40–60 μm size (Fig. 1-b).

Pancreatic cancer cells, PANC-1, MIA PaCa-2, and AsPC-1, exhibited weak aggregation resulting in smaller spheroids with higher dissemination patterns under mono-culture condition (Fig. 1-c). Among three cell lines, only PANC-1 cells formed compact spheroids and about 30–40% of spheroids showed invasive boundaries with peripheral protrusions. The other two pancreatic cancer cell lines, MIA PaCa-2, and AsPC-1, formed loose aggregates. In contrast, hepatic (Huh-7) and colorectal (HT-29) cancer cells formed relatively larger and highly compact aggregates with smooth boundaries.

### Activation of PSCs by co-culturing with tumor spheroids

**Fig. 2:**
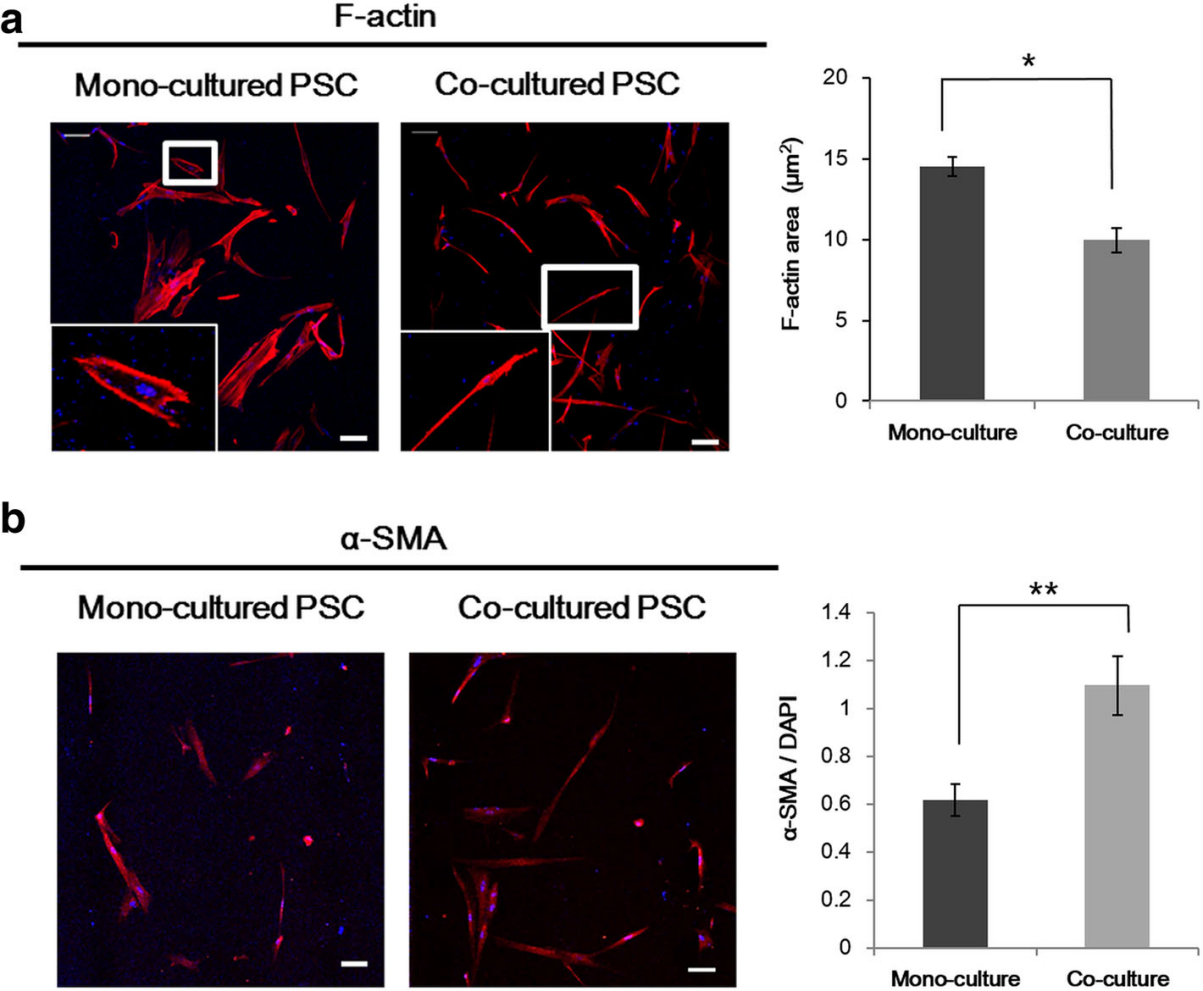
Activation of PSCs co-cultured with 3D tumor spheroids. Changes in morphology (**a**) and α-SMA expression level (**b**) of PSCs when co-cultured with PANC-1 tumor spheroids. Cells were grown for 5 days before all measurements. Optical sections were acquired at 6 μm intervals and stacked into a z-projection from which fluorescence intensity was calculated. Data are expressed as the mean ± SE of 3 independent experiments. Scale bars, 100 μm; Student’s t-test, *p < 0.05, **p < 0.01. PSCs, pancreatic stellate cells; α-SMA, alpha smooth muscle actin

PSCs were cultured as embedded in the collagen gel and their growth and morphology was monitored over 5 days. When PSCs were cultured with PANC-1 tumor spheroids for 5 days, a prominent change in cell morphology was observed upon staining with F-actin (Fig. 2-a). Under co-culture condition, PSCs assumed an elongated spindle shape resulting in a significant decrease in F-actin area, indicating their activation (14.53 μm^2^ vs 9.95 μm^2^). PSC activation under co-culture conditions was also demonstrated by the increased expression of α-SMA, which is a marker of activated stellate cells (Fig. 2-b).

### Increased cell migration under co-culture condition

**Fig. 3:**
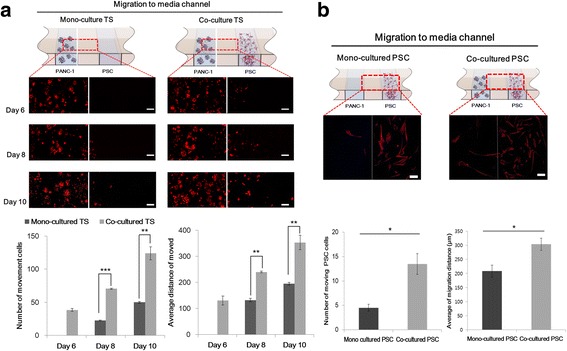
Increased migration of cancer cells and PSCs. **a** Increased migration of cancer cells out of the spheroid channel. Representative images of PKH26-labelled cancer cells migrating into the media channel are shown at days 6, 8, and 10. **b** Increased migration of PSCs (stained with F-actin) towards the tumor spheroids compartment after 5 days of culture. The location of PSCs was determined using nucleus staining. Optical sections were acquired at 6 μm intervals and stacked into a z-projection. Data are expressed as the mean ± SE of 3 independent experiments. Scale bars, 100 μm; Student’s t-test, *p < 0.05, **p < 0.01, ***p < 0.001. PSCs, pancreatic stellate cells; TS, tumor spheroids

The effect of activated PSCs on the migratory ability of cancer cells was determined by measuring the movement of cancer cells under mono- and co-culture condition on days 6, 8 and 10 (Fig. 3-a). While mono-cultured spheroids did not show notable migration of cells on day 6, spheroids co-cultured with PSCs showed significant cell migration, indicating the stimulatory effect of PSCs on the onset and progress of cancer cell movement. On day 8 and 10, migratory ability of cancer cells increased by about 2–3 folds in terms of cell numbers and distance moved under PSC co-culture condition (Fig. 3-a). Upon co-culturing with cancer cells, PSC activation was also evident in regards to morphological changes and increased migration toward tumor spheroids. On day 5, PSCs were found in significantly greater numbers in the media channel and migrated longer distances when co-cultured with tumor spheroids (Fig. 3-b). Overall these results indicated that mutual activation between cancer cells in spheroids and PSCs resulted in increased invasion and migration of both cell types in our microchannel system.

### Increased expression of EMT markers in tumor spheroids under co-culture

**Fig. 4:**
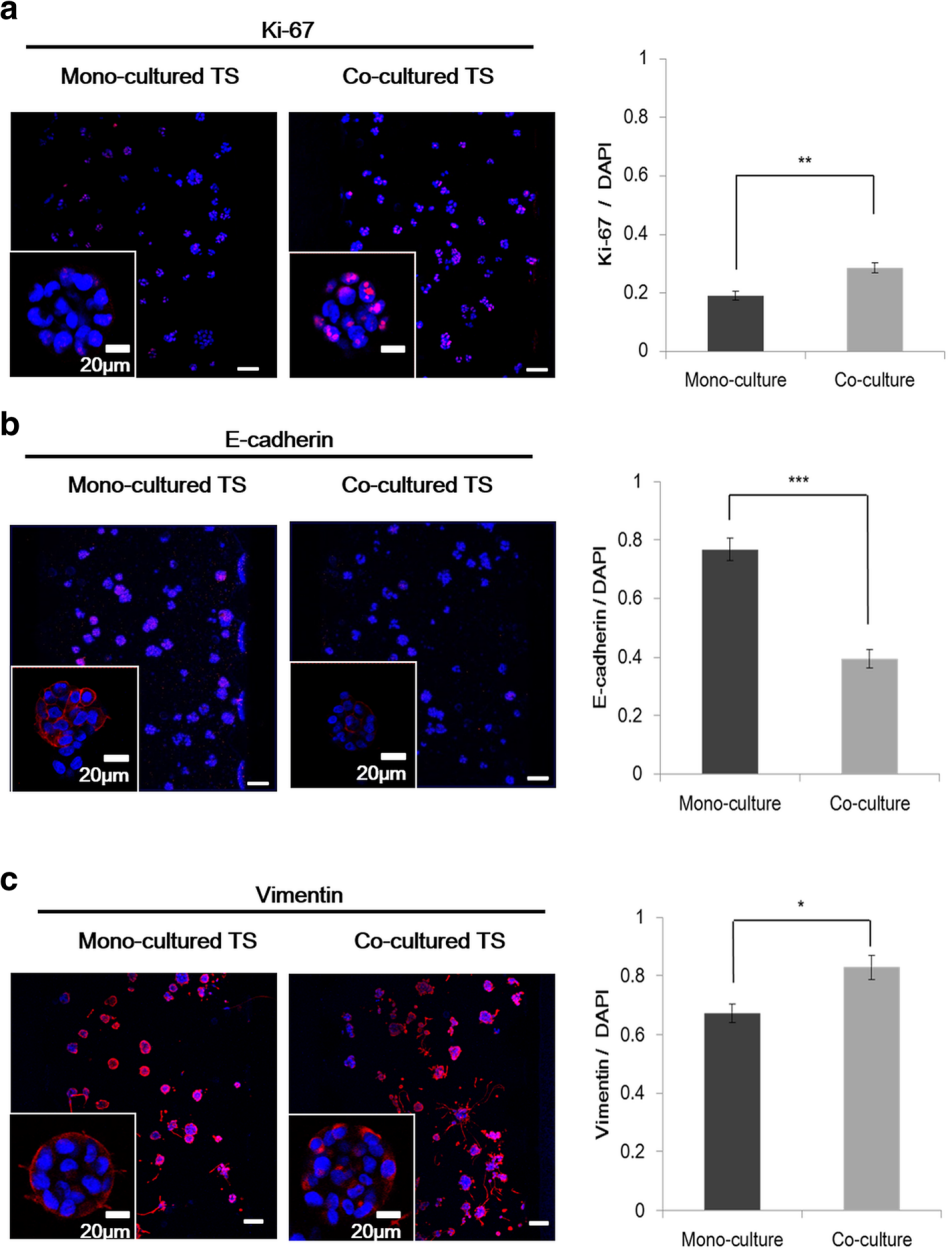
Expression of EMT-related markers in PANC-1 tumor spheroids. Significant changes in the expression level of three proteins related to EMT are shown for Ki-67 (**a**), E-cadherin (**b**) and vimentin (**c**) upon co-culture with PSCs. Spheroids were cultured for 5 days. Optical sections were acquired at 6 μm intervals and stacked into a z-projection from which fluorescence intensity was calculated. Data are expressed as the mean ± SE of 3 independent experiments. Scale bars 20 μm, 100 μm. Student’s t-test, *p < 0.05, ** *p* < 0.01, *** *p* < 0.001. PSCs, pancreatic stellate cells; TS, tumor spheroids; EMT, epithelial-mesenchymal transition

In order to examine the molecular mechanisms associated with the increased proliferation and migration of cancer cells, expression of EMT-related proteins was evaluated (Fig. 4). In agreement with the increased number of spheroids (Fig. 1-a, b), Ki-67, a proliferation marker, showed increased expression in tumor spheroids co-cultured with PSCs (Fig. 4-a). The expression of E-cadherin was quite low in PANC-1 cells grown in 3D spheroids and became undetectable when co-cultured with PSCs (Fig. 4-b). It was also noteworthy that vimentin expression was very high in PANC-1 cancer cells grown in 3D spheroids and further increase was observed upon PSC co-culture (Fig. 4-c).

### Differential protein expression in tumor spheroids upon co-culture with PSCs

**Fig. 5:**
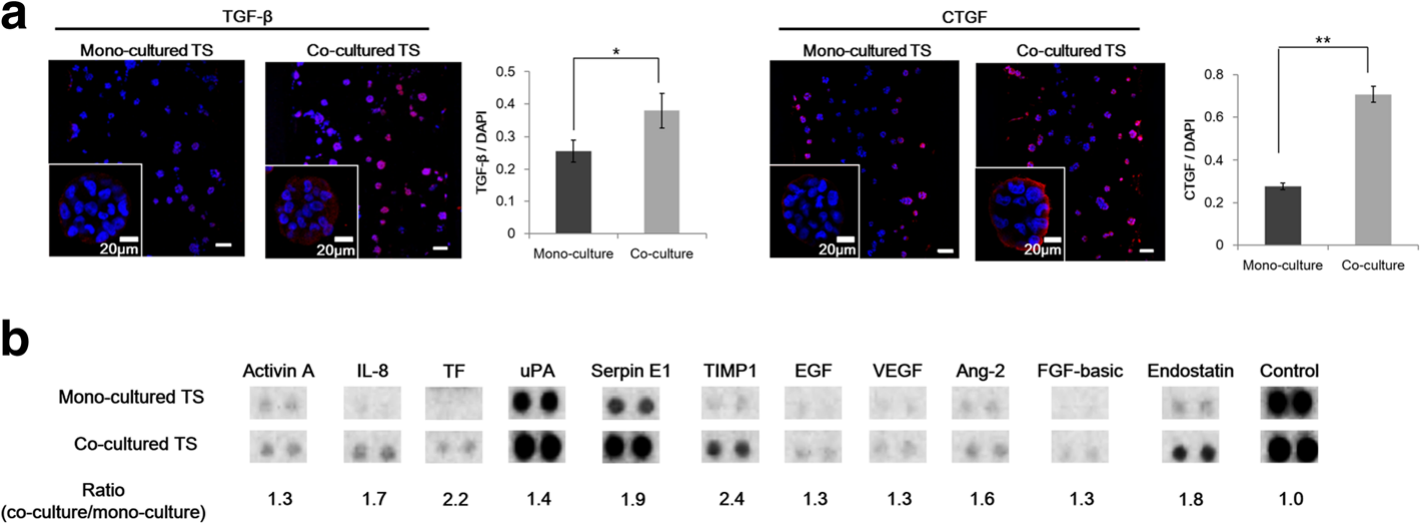
Expression of cytokines in PANC-1 tumor spheroids under co-culture conditions. **a** PANC-1 tumor spheroids were cultured for 5 days and stained for TGF-β, and CTGF. Optical sections were acquired at 6 μm intervals and stacked into a z-projection from which fluorescence intensity was calculated. Data are expressed as the mean ± SE of 3 independent experiments. **b** PANC-1 spheroids were grown for 5 days and harvested for proteome analysis using Proteome Profiler™. Duplicate measurements were performed to assess reproducibility of the data. Scale bars 20 μm, 100 μm. Student’s t-test, * *p* < 0.05, *** *p* < 0.001. TGF-β, transforming growth factor beta; CTGF, connective tissue growth factor; TS, tumor spheroids

Proteome analysis was performed to detect changes in cytokine expression in tumor spheroids upon PSC co-culture. Transforming growth factor beta (TGF-β) and connective tissue growth factor (CTGF) showed increased expression upon co-culture with PSCs, indicating their association with migration and tumor growth shown above (Fig. 5-a). Expression of many factors was significantly higher in co-cultured condition (Fig. 5-a). Among the 55 different cytokines tested, 11 factors showed significant changes with differences exceeding 1.3-fold; these factors included tissue inhibitor of metalloproteinase 1 (TIMP-1), tissue factor/factor III (TF), and serpin E1 (PAI-1), collagen XVIII/endostatin (Fig. 5-b).

### Drug sensitivity of PANC-1 tumor spheroids induced by PSCs co-culturing

**Fig. 6:**
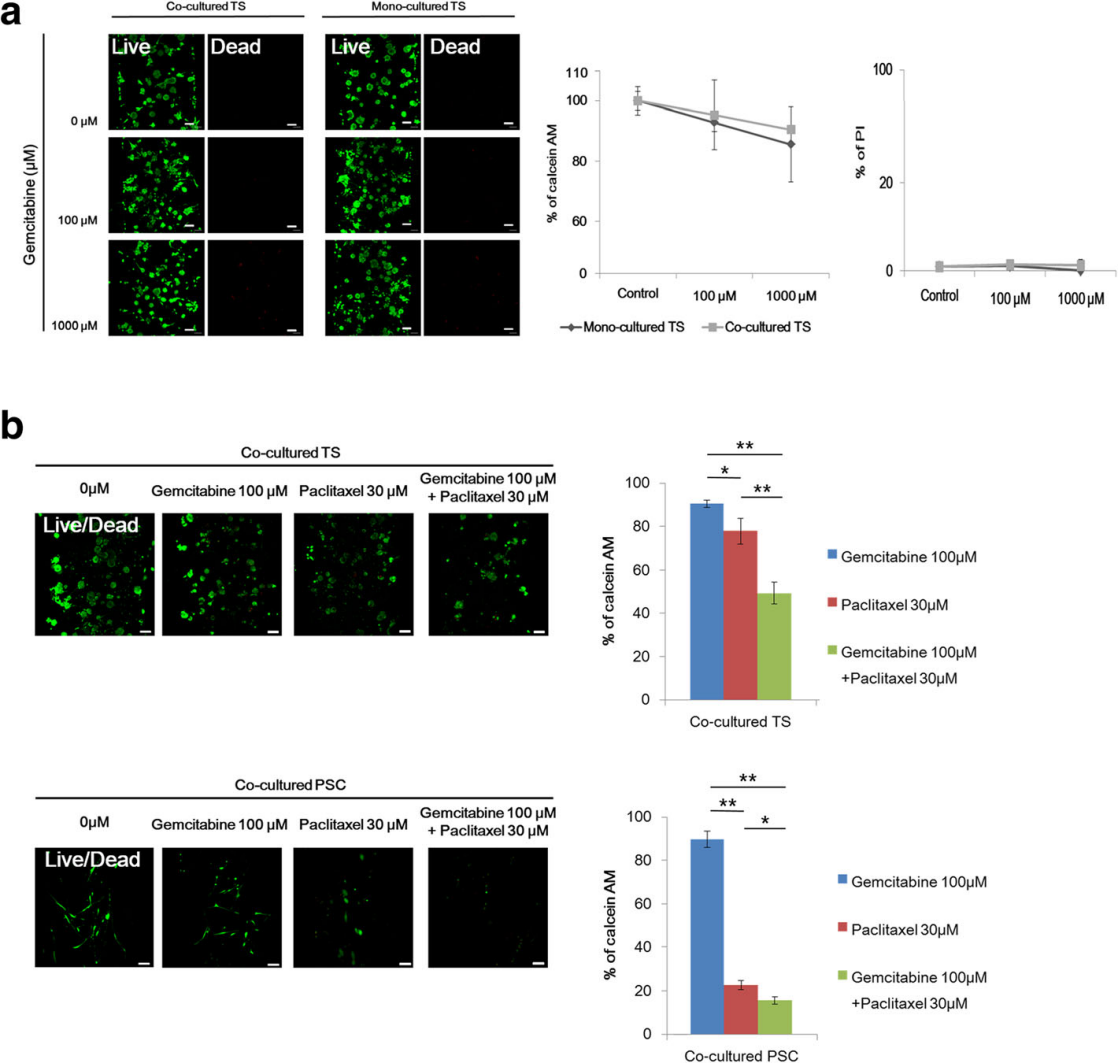
Differential sensitivity of PANC-1 tumor spheroids to anti-cancer drugs. Cells were grown for 5 days and stained for viability (calcein AM/PI) after 72 h exposure to gemcitabine (**a**), combination of gemcitabine and paclitaxel (**b**). Optical sections were acquired at 6 μm intervals and stacked into a z-projection from which fluorescence intensity was calculated. Data are expressed as the mean ± SE of 3 independent experiments. Scale bars 100 μm. Student’s t-test, *p < 0.05, ** *p* < 0.01. PSCs, pancreatic stellate cells; TS, tumor spheroids

It has been reported that mechanisms involved in EMT also contribute to drug resistance. Hence we compared drug sensitivity in PANC-1 spheroids cultured with or without PSCs. No significant changes in viability were shown for either mono- or co-cultured tumor spheroids exposed to gemcitabine; almost 80–90% of tumor cells remained viable at drug concentrations up to 1 mM (Fig. 6-a). Oxaliplatin produced cell-killing effect in a dose dependent manner (Additional file 1: Figure S1-a). On the other hands, PSCs showed resistance to both oxaliplatin and gemcitabine showing over 80% survival when exposed to 100 μM, although a reduction in viability was obtained at even higher doses exceeding 300 μM (Additional file 1: Figure S1-b). We tested the potential synergism between gemcitabine and paclitaxel and found their synergistic interaction as supra-additive reduction in viability, i.e., 50% viability reduction in combination vs. only 10% and 20% reduction for each drug alone (Fig. 6-b). This synergy can be attributed to the greater sensitivity of PSCs to paclitaxel compared to other anti-cancer drugs (Fig. 6-b, Additional file 1: Figure S1-b, c).

## Discussion

The purpose of 3D culture models for human solid tumors is to reproduce in vivo like characteristics in terms of in cellular morphology, behavior, cell-cell and cell-matrix interactions. There are several available 3D cell culture platforms such as the hanging drop method, liquid overlays, microwell arrays, cellular microarrays on a functionalized glass slide, micropillar arrays and microfluidic devices [22, 33]. Pancreatic 3D tumor spheroids have been developed using nano-patterned-bottom plates or a hanging drop method [34, 35]. We previously reported pancreatic tumor spheroid formation using microwell and micropillar arrays [33, 36]. In this study, we developed a 3D system of co-culturing pancreatic tumor spheroids and stromal cells using microchannel plates, which mimics the tumor microenvironmental interaction. The main in vivo-like features of our co-culture model are: 1) formation of multicellular tumoroid of pancreatic cancer cells which otherwise do not usually form stable aggregates; 2) accommodation of three dimensionality by embedding cancer cells and PSCs in collagen gel stroma; 3) presence of heterotypic cell interactions between cancer cells and PSCs via paracrine mechanism.

We designed a microfluidic channel plate to recapitulate the in vivo reciprocal interaction of cancer cells-PSCs in a miniature scale and to be able to perform multiplexed cell staining assay for high content imaging (HCI). Macroscale 3D culture systems such as hanging drop methods are recognized for their disadvantages in HCI assays, which include low throughput of culturing and imaging [22]. Microfluidic platforms were useful in miniaturizing cultures while maintaining 3D embedment in bio matrix collagen. These platforms have three cell loading channels; a central channel for cancer cells and two others for PSCs (Fig. 1-a). Side channels can be used for mono- vs. co-culture comparisons within one chip by loading PSCs in only one of the side channels. Channel dimension of 1000 μm width and 190 μm height provided media volume and spatial condition enough for long term culture as well as spheroid growth [28], while ensuring a short distance for cellular interaction via soluble factors compared to the conditions provided by conventional transwell methods. Each channel was assigned to different cell types to study paracrine interactions and to monitor or harvest each cell types for molecular expression profiles or viability. Our design can also be applied to studies of juxtacrine interaction by loading both cell types into the same channel. Our migration assay measure the number of cells migrating out of the channels following their invasive movement through the ECM, and the distance moved subsequently (Fig. 4). These conditions recapitulates metastasis more closely than those of conventional wound healing or Boyden chamber assays.

The prominent roles of PSCs in the growth and progression of PDAC are well reported [4, 5]. Following activation, PSCs undergo major morphological and functional changes and induce enhanced proliferation of cancer cells and ECM deposition leading to aggressive tumor growth and desmoplasia. Activated PSCs are also known to secrete many paracrine factors stimulating migration of cancer cells and ECM remodeling, which occurs in the invasive front of pancreatic tumors [37]. Highly activated PSCs were evident in our 3D culture model (Fig. 2): PSCs cultured three dimensionally by embedding in a collagen matrix, underwent morphological changes from a relatively short and stellate shape to a long spindle shape with stress fiber formation (Fig. 2-a). Further changes to a highly elongated shape with increased expression of α-SMA (a commonly used and reliable marker for activated PSCs) were observed under the influence of co-cultured tumor cells (Fig. 2), indicating additional changes in the activated state of PSCs. Factors such as thrombospondin, urokinase-type plasminogen activator (uPA), epithelial growth factor (EGF), serpin E1, matrix metalloproteinases (MMPs) and dipeptidylpeptidase IV (CD 26) belong to the secretome of activated PSCs that promotes tumor invasion [37–39]. Several of these factors among these, such as CD26, thrombospondin, uPA, and serpin E1 were found to be highly expressed in 3D–cultured PSCs (Additional file 2: Figure S2), indicating that these factors may be responsible for the cancer cell and PSC migration (Fig. 3-a, b). Increased migration of PSC was observed when co-cultured with cancer cells, yet the expression level of these factors did not change, suggesting that there are other factors involved with PSC self-motility [37, 38]. Overall, these results indicate the utility of our in vitro microchannel 3D co-culture model for studying the activation of PSCs and their roles in EMT and migration of pancreatic cancer cells in the invasive front of human tumor tissues in vivo.

Pancreatic cell lines such as PANC-1, AsPC-1, Capan-2 and MIA PaCa-2, usually show limited tendency to aggregate, hence, they do not form multicellular spheroids by using conventional methods such as liquid overlay technique [35, 36, 40–42]. A miniaturized culturing method using concave microwells was useful in spheroid formation of PANC-1 cells but not for the other cell lines [43]. Culturing 3D as embedded in collagen matrix using microchannel plates facilitated spheroid formation of all the pancreatic cancer cell lines except Capan-1 and Capan-2 (Fig. 1-c). PANC-1 spheroids formed in collagen matrix appeared smaller in size with a rough surface of protruding cells (Fig. 1-c) in contrast to those of HT-29 and Huh-7 cells which have greater aggregating properties to form compact spheroids under simple low attachment condition without any other aids [28, 36]. In addition, PANC-1 spheroids showed particularly low level of E-cadherin and high expression of vimentin, which is consistent with results reported by others [44], and also in contrast to HT-29 and Huh-7 cells (Additional file 3: Figure S3). The morphology and structure of spheroids as well as mesenchymal marker expression of PANC-1 cells was consistent with their increased invasive motility within collagen matrix (Fig. 3-a).

Motility of PANC-1 cells was increased under PSC co-culture condition as shown by the increased number of cells migrating out of the collagen matrix and into the media channel (Fig. 3-a). Concurrent changes were seen in the expression levels of EMT markers (Fig. 4). This change was also accompanied by the increased expression of well-known EMT inducers such as TGF-β and CTGF as well as several other factors including activin A, interleukin-8 (IL-8), tissue factor (TF), uPA, serpin E1, and TIMP1 as determined by immunofluorescence staining and proteome assay (Fig. 5). TGF-β acts not only as a inducer of cell proliferation, EMT, and invasion, but also as a key mediator of the interaction between cancer and stellate cells leading to a desmoplastic and immunosuppressive environment in PDAC [45]. CTGF functions as a potential driver of EMT and desmoplasia in TGF-β-dependent as well as independent pathways, hence, representing a unique target in pancreatic cancer [46]. Activin A is a member of the TGF-β superfamily and known for its context-dependent signaling in cancer cell invasion [47, 48]. IL-8, which is a pro-inflammatory cytokine produced by either cancer cells or fibroblasts, plays a critical role in EMT and invasiveness [49]. TF is involved in remodeling of the cytoskeleton and tumor microenvironment, enhancing cell migration and metastasis [50]. uPA and serpin E1 are involved in tumor migration via their complex signaling network with TGF-β [51, 52]. TIMP-1 overexpression has been consistently associated with cancer progress and recently reported to induce an EMT phenotype and to mediate cancer-stromal cell crosstalk, independent of its MMP-inhibitory function [28, 32, 53]. Among the other upregulated factors such as EGF, vascular endothelial growth factor (VEGF), angiopoietin-2 (Ang-2), and basic fibroblast growth factor (bFGF), are all well-known for their tumor promoting or EMT-inducing effects, except endostatin [54, 55]. PSC-mediated increases in expression of collagen XVIII/endostatin can be a compensatory event against the upregulation of angiotensin-2 directing angiogenesis. It can also be speculated, however, that increased level of collagen XVIII/endostatin could contribute to EMT of PANC-1 cells stimulating cell motility. Despite the antiangiogenic activity reported for endostatin, other studies have provided conflicting data. Serum endostatin levels were found to be elevated and correlated with VEGF levels in cancer patients [56], and collagen XVIII and its fragment, endostatin was suggested to have a role in EMT during cardiac development [57]. It is noteworthy that a group of factors upregulated in our 3D matrix-embedded tumor spheroids upon PSC co-culture are the ones reportedly known for their tumor promoting and EMT inducing activity in PDAC and other types of cancers. This strongly supports the validity of our 3D co-culture as a clinically-relevant model as well as its utility in evaluating novel microenvironmental targets and anticancer agents in vitro.

Resistance to apoptotic stimuli such as drug treatment is one of the functional implications of EMT along with enhanced mobility and invasion [58]. The contribution of EMT to drug resistance has been reported [16] and since then, it has been validated in many other studies not only for PDAC but also for metastatic breast cancer models [59], providing a rationale for therapeutic targeting of EMT. When compared with colorectal HT-29 spheroids, PANC-1 spheroids showed greater cell motility along with significantly higher vimentin and lower E-cadherin expression (Fig. 4, data not shown) [28]. Upon cancer-associated fibroblast (CAF) or PSC co-culture, HT-29 spheroids showed increased drug resistance [28], whereas PANC-1 spheroids showed no further changes in drug response, which could be associated with the intrinsically advanced status of EMT (Fig. 6-a). Albumin-bound paclitaxel (Nab-Paclitaxel) in addition to gemcitabine has shown clinical improvement [2, 60] and the synergy of this combination treatment was attributed not only to the direct cytotoxic effect of paclitaxel on cancer cells, but also depletion of the peritumoral desmoplastic stroma and increased intratumoral gemcitabine levels [2]. Selective binding of Nab-Paclitaxel to the extracellular matrix protein, SPARC (Secreted Protein Acidic and Rich in Cysteine) [61] or macrophage activation via micropinocytosis [62] have been suggested as mechanism behind the selective response. We found the combination of gemcitabine and paclitaxel to be highly synergistic, which was attributed to the selective inhibitory effect of paclitaxel on the viability of PSC (Fig. 6-b). The effect of paclitaxel on PSC was obvious at a concentration as low as 30 µM, as compared to the concentration of oxliaplatin and gemcitabine (Additiona file 1: Figure S1-b). Although paclitaxel is known to inhibit microtubule dynamics by stabilizing it, it also inhibits actin polymerization due to interactions between the microtubule system and the actin cytoskeleton which occurs via mechanical coupling and other mechanisms [43]. Hence, paclitaxel has been shown to inhibit stress fiber formation and myofibroblast differentiation induced by TGF-β [43]. In addition, stimulation of fibroblast contractility and actin organization by microtubule poisons (e.g., vinblastine) was found to be prevented by paclitaxel [63] and indirect effects of paclitaxel on intermediate filaments in fibroblasts has also been reported [64]. Overall these data suggest that our model can be used to study not only EMT-related phenotypic change but also drug resistance mediated by microenvironmental factors, such as stromal cells and their interaction.

Contradictory results obtained using different CAF targeting strategies have raised the issue of intratumoral CAF heterogeneity via distinguishable mechanisms of activation such as juxtacrine (contact-dependent) or paracrine manner [13]. Using our microchannel model, it was demonstrated that PSC activation by tumor cells could be effective over 1 mm distance, suggesting that CAF recruitment may occur over an extensive region around the tumor mass. This PSC activation in turn should results in increased migration of tumor cells via reciprocal activation. Our model has also proven its usefulness in drug evaluation where the differential sensitivity of different cell types can be determined and optimization of drug combination regimens can be studied.

## Conclusions

In summary, we developed a 3D pancreatic tumor model in vitro by co-culturing pancreatic tumor spheroids with PSC in a collagen matrix, under these conditions we observed cancer cell-cell interactions, cell-ECM interactions, and cancer cell-PSC interactions. These conditions were generated in a microchannel (microfluidic) plate which accommodated sub-millimeter interactions between adjacent cell types, mimicking in vivo tumor microenvironmental interactions. The 3D reciprocal activation between the two cell types was confirmed by enhanced cell growth, increased cell motility, expression of EMT-related factors, and drug resistance. Our microfluidic co-culture of pancreatic tumor spheroids with PSC may be a valuable tool for studying EMT and drug resistance under in vitro conditions that simulated the in vivo tumor microenvironment.

## Additional files

Additional file 1: Figure S1. Differential sensitivity of PANC-1 tumor spheroids and PSCs to gemcitabine and oxaliplatin. Cells were grown for 5 days and stained with calcein AM / PI after 72 h exposure to oxaliplatin (a, b) and gemcitabine (c) under mono- or co-culture condition. Optical sections were acquired at 6 μm intervals and stacked into a z-projection from which fluorescence intensity was calculated. Data are expressed as the mean ± SE of 3 independent experiments. Scale bars 100 μm. Data showed no significance from Student’s t-test. No statistically significantdifferences were observed. PSCs, pancreatic stellate cells; TS, tumor spheroids. (TIFF 6300 kb)

Additional file 2: Figure S2. Expression of cytokines in PSCs. (a) PSCs were grown for 5 days with or without PANC-1 spheroids in microchannel plate and harvested for proteome analysis using Proteome Profiler™. PSCs, pancreatic stellate cells; TS, tumor spheroids. (TIFF 2828 kb)

Additional file 3: Figure S3. Differential expression of EMT-related markers in different tumor cell spheroids. Immunofluorescence staining of E-cadherin and vimentin was performed in PANC-1 and HT-29 spheroids cultured for 5 days in microfluidic channels, and on paraffin sections of Huh-7 spheroids cultured for 5 days in ULA 96 well plates. For PANC-1 and HT-29 spheroids (red), confocal optical sections were acquired at 2 μm intervals and stacked into a z-projection (see Methods for details). Counter stain, DAPI (blue). Scale bars, 20 μm and 100 μm. EMT, epithelial-mesenchymal transition; TS, tumor spheroids. (TIF 667 kb)
