# Supplementary figures and images for: Microfluidic co-culture of pancreatic tumor spheroids with stellate cells as a novel 3D model for investigation of stroma-mediated cell motility and drug resistance
Source: J Exp Clin Cancer Res. 2018 Jan 12;37:4. doi: 10.1186/s13046-017-0654-6 (PMC5767067; doi:10.1186/s13046-017-0654-6)

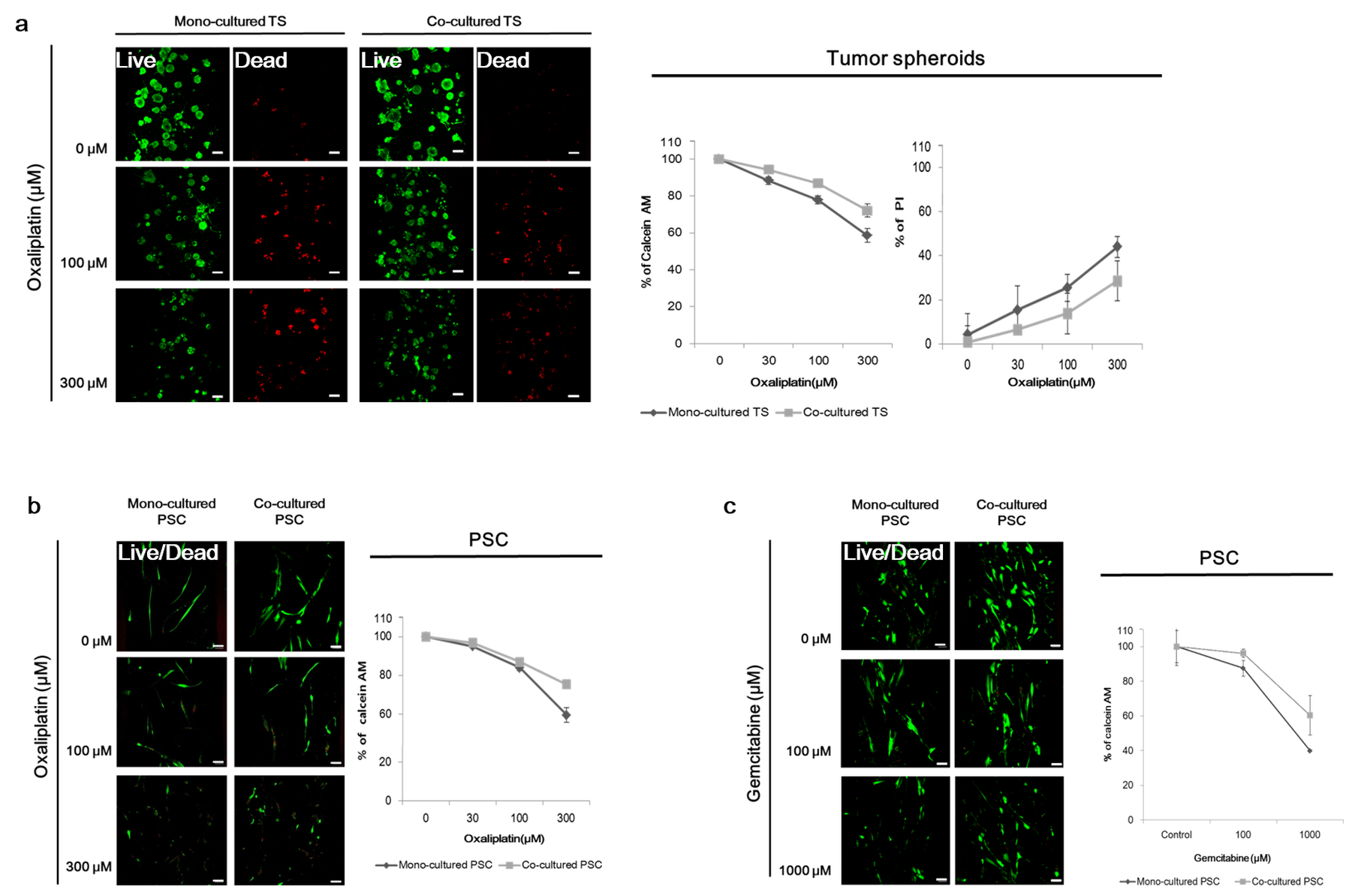

Supplement: Supplementary file 1 — Differential sensitivity of PANC-1 tumor spheroids and PSCs to gemcitabine and oxaliplatin. Cells were grown for 5 days and stained with calcein AM / PI after 72 h exposure to oxaliplatin (a, b) and gemcitabine (c) under mono- or co-culture condition. Optical sections were acquired at 6 μm intervals and stacked into a z-projection from which fluorescence intensity was calculated. Data are expressed as the mean ± SE of 3 independent experiments. Scale bars 100 μm. Data showed no significance from Student’s t-test. No statistically significantdifferences were observed. PSCs, pancreatic stellate cells; TS, tumor spheroids. (TIFF 6300 kb) [file 13046_2017_654_MOESM1_ESM.tif]

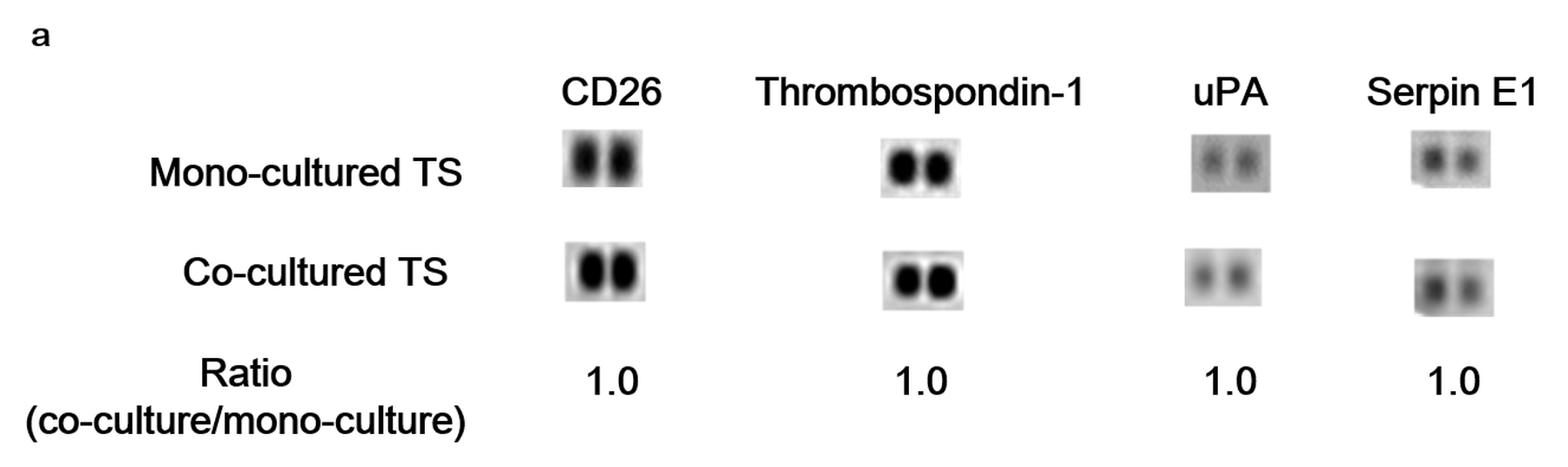

Supplement: Supplementary file 2 — Expression of cytokines in PSCs. (a) PSCs were grown for 5 days with or without PANC-1 spheroids in microchannel plate and harvested for proteome analysis using Proteome Profiler™. PSCs, pancreatic stellate cells; TS, tumor spheroids. (TIFF 2828 kb) [file 13046_2017_654_MOESM2_ESM.tif]

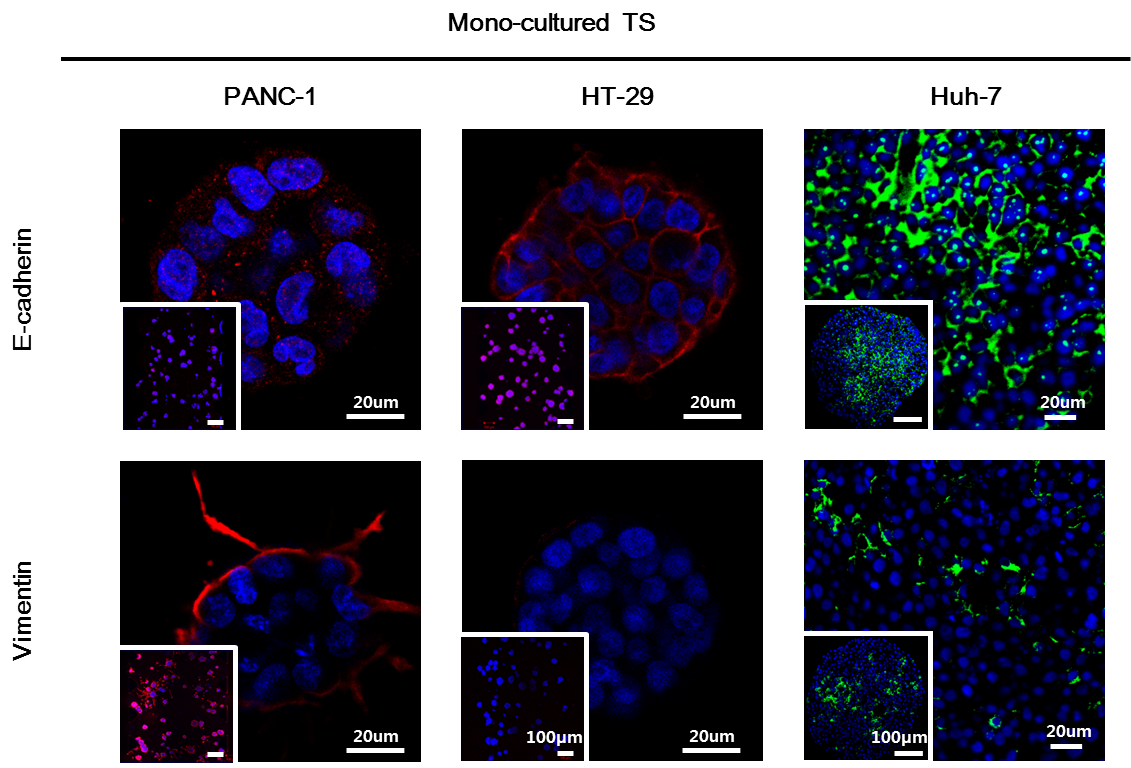

Supplement: Supplementary file 3 — Differential expression of EMT-related markers in different tumor cell spheroids. Immunofluorescence staining of E-cadherin and vimentin was performed in PANC-1 and HT-29 spheroids cultured for 5 days in microfluidic channels, and on paraffin sections of Huh-7 spheroids cultured for 5 days in ULA 96 well plates. For PANC-1 and HT-29 spheroids (red), confocal optical sections were acquired at 2 μm intervals and stacked into a z-projection (see Methods for details). Counter stain, DAPI (blue). Scale bars, 20 μm and 100 μm. EMT, epithelial-mesenchymal transition; TS, tumor spheroids. (TIF 667 kb) [file 13046_2017_654_MOESM3_ESM.tif]
